# Plant-Forward Dietary Patterns and Cardiometabolic Risk Among US Adults: Findings From the National Health and Nutrition Examination Survey, 2009–2023

**DOI:** 10.5888/pcd23.260232

**Published:** 2026-11-05

**Authors:** Darren Lov

**Affiliations:** 1 UCLA Fielding School of Public Health, Department of Health Policy and Management, Los Angeles, California

## Abstract

**Objective:**

The objective of this study was to describe the relationship between plant-forward dietary patterns and metabolic profiles.

**Methods:**

A repeated cross-sectional analysis of adults aged 18 years or older was conducted using data from the National Health and Nutrition Examination Survey (NHANES), 2009–2023. Plant-forward dietary adherence was estimated from the mean of two 24-hour dietary recalls and categorized into tertiles. Survey-weighted multivariable regression examined associations with body mass index (BMI), obesity, prediabetes, and diabetes, adjusting for demographic, socioeconomic, dietary, and behavioral factors.

**Results:**

Among 33,532 NHANES participants, greater plant-forward dietary adherence was associated with lower BMI (β = −1.45; 95% CI, −1.82 to −1.09), lower odds of obesity (adjusted odds ratio [AOR] = 0.70; 95% CI, 0.62–0.79) and diabetes (AOR = 0.72; 95% CI, 0.62–0.84) but not prediabetes (AOR = 0.92; 95% CI, 0.82–1.04).

**Conclusion:**

Greater adherence to plant-forward dietary patterns was associated with lower BMI and reduced odds of obesity and diabetes among US adults.

**Summary**

**What is already known on this topic?**

Plant-forward dietary patterns are associated with favorable cardiometabolic health, but evidence from nationally representative US populations is limited.

**What is added by this report?**

Among 33,532 US adults, greater plant-forward dietary adherence showed graded associations with lower body mass index and obesity and was independently associated with lower odds of diabetes after accounting for demographic, socioeconomic, dietary, and behavioral factors.

**What are the implications for public health practice?**

Population-level strategies that increase access to and support consumption of minimally processed plant foods may complement existing efforts to reduce the prevalence of cardiometabolic disease.

## Objective

Cardiometabolic conditions, including obesity and diabetes, are leading contributors to illness and death in the US, with persistent disparities across socioeconomic and structural contexts (1). Dietary patterns are important determinants of cardiometabolic health (2), and plant-forward dietary patterns, which emphasize minimally processed plant foods while allowing moderate amounts of animal-source foods (3), have been associated with lower body weight and more favorable metabolic profiles (4). Despite increasing evidence supporting plant-forward dietary patterns, current US dietary recommendations emphasize dietary patterns comprising relatively high consumption of animal-source foods, particularly red meat (5). However, population-level evidence examining the cardiometabolic implications of more plant-forward dietary patterns among US adults is limited.

Using nationally representative data from the National Health and Nutrition Examination Survey (NHANES) for 2009 through 2023, the association between a nutrient-derived proxy of adherence to plant-forward dietary patterns and cardiometabolic outcomes was examined among US adults. It was hypothesized that greater adherence to a nutrient-derived proxy of a plant-forward dietary pattern would be associated with more favorable cardiometabolic profiles, including lower body mass index (BMI) and reduced odds of obesity, prediabetes, and diabetes.

## Methods

### Study design and population

A repeated cross-sectional study of adults aged 18 years or older used publicly available 2009–2023 NHANES data. NHANES uses a complex, multistage probability sampling design to represent the noninstitutionalized US adult population (6). Analyses were restricted to participants with complete data for variables included in the regression models. Consequently, analytic sample sizes varied across outcomes according to data availability.

### Outcome variables

Cardiometabolic outcomes included BMI, obesity, prediabetes, and diabetes, and were assessed by using standardized examination and laboratory procedures. BMI was calculated from measured height and weight (kg/m^2^) and analyzed continuously; obesity was defined as BMI of 30 or more. Prediabetes was defined as glycated hemoglobin A_1c_ (HbA_1c_) of 5.7% to 6.4%, excluding people with diabetes. Diabetes was defined by self-reported physician diagnosis or an HbA_1c_ of 6.5% or more, capturing both diagnosed and undiagnosed disease (7).

### Exposure

The primary exposure was a study-specific nutrient-derived proxy for adherence to a plant-forward dietary pattern derived from the mean of two 24-hour dietary recalls (plant-forward score). Participants were assigned scores for mean intake of daily fiber, saturated fat, and cholesterol, with fiber scored positively and saturated fat and cholesterol reverse-scored, representing dietary characteristics consistent with plant-forward eating. Fiber indicated plant food intake, whereas cholesterol and saturated fat were included because plant-forward dietary patterns generally, although not universally, contain lower amounts of these nutrients. Component scores were summed (range, 0–6), with higher values indicating greater adherence, and categorized into tertiles for regression analyses. Modeling dietary adherence as tertiles enabled assessment of graded associations.

### Covariates

Covariates included age group (18–29, 30–49, 50–64, ≥65 y), sex (female, male), race and ethnicity (Mexican or Hispanic, non-Hispanic Black, non-Hispanic White, Other or multiracial), tertile of poverty-to-income ratio, food security (very low, low, marginal, high), physical activity in the past week (0, <150, 150–299, or ≥300 min/week), total energy intake per 100 kcal per day, and survey cycle (2009–2010, 2011–2012, 2013–2014, 2015–2016, 2017–2020, 2021–2023).

### Statistical analysis

Survey-weighted descriptive statistics were used to characterize the sample. Survey-weighted multivariable linear and logistic regression models assessed associations with BMI, obesity, prediabetes, and diabetes. Models were adjusted for all covariates. Significance was defined as a 2-sided *P* value less than .05. Analyses were performed using R version 4.2.2 (R Core Team).

## Results

The analytic sample included 33,532 NHANES participants. Survey-weighted analyses produced estimates representative of the US civilian noninstitutionalized adult population (Table 1). Participants were 51.7% female, and the racial and ethnic composition was 64.5% non-Hispanic White, 15.3% Mexican or Hispanic, 11.5% non-Hispanic Black, and 8.7% Other or multiracial. Overall, 38.5% had obesity, 20.9% had prediabetes, 12.7% had diabetes, and mean (SD) BMI was 29.3 (7.1).

**Table 1:** Survey-Weighted Characteristics of Survey Participants by Tertile of Plant-Forward Dietary Score,^a^ NHANES, 2009–2023^b^

Characteristic	Overall (N = 33,532)	Tertile of plant-forward dietary score^a^			*P* value^c^
		Low adherence (n = 11,093)	Moderate adherence (n = 11,139)	High adherence (n = 11,300)	
**Prediabetes**					
No	79.1	78.6	79.7	79.1	.40
Yes	20.9	21.4	20.3	20.9	
Missing	3.0	2.9	2.9	3.1	
**Diabetes**					
No	87.3	87.0	87.4	87.4	.80
Yes	12.7	13.0	12.6	12.6	
Missing	1.6	1.5	1.6	1.9	
**Obesity**					
No	61.5	58.3	60.8	65.5	<.001
Yes	38.5	41.7	39.2	34.5	
Missing	0.8	0.6	0.9	1.1	
**Body mass index (not weighted)^d^**					
Mean (SD)	29.3 (7.1)	29.9 (7.2)	29.3 (7.1)	28.6 (6.7)	<.001
Missing	0.8	0.6	0.9	1.1	
**Food security level**					
Very low	7.1	7.5	6.8	7.1	.04
Low	8.6	9.2	8.3	8.3	
Marginal	10.0	10.6	10.3	9.2	
High	74.2	72.7	74.6	75.5	
Missing	2.6	2.2	2.8	2.8	
**Age group, y**					
18–29	20.9	22.8	21.7	18.0	<.001
30–49	33.9	35.8	33.8	32.1	
50–64	27.6	26.9	26.8	29.4	
≥65	17.5	14.5	17.7	20.5	
**Sex**					
Male	48.3	60.2	47.9	36.3	<.001
Female	51.7	39.8	52.1	63.7	
**Race and ethnicity**					
Mexican or Hispanic	15.3	14.1	16.4	15.3	<.001
Non-Hispanic Black	11.5	13.2	10.8	10.3	
Non-Hispanic White	64.5	66.0	64.5	63.0	
Other or multiracial^e^	8.7	6.6	8.2	11.4	
**Tertile of poverty-to-income ratio**					
First tertile (lower income relative to poverty threshold)	23.4	23.5	23.8	22.9	.12
Second tertile	31.0	31.8	31.4	29.8	
Third tertile (higher income relative to poverty threshold)	45.6	44.7	44.8	47.4	
Missing	8.1	7.1	8.4	8.9	
**Physical activity in past week, in minutes per week**					
0	39.5	44.5	37.4	36.3	<.001
<150	18.4	17.2	19.1	19.0	
150–299	13.5	11.5	13.5	15.5	
≥300	28.7	26.9	30.0	29.2	
Missing	0.3	0.2	0.3	0.2	
**Energy intake per 100 kcal per day (not weighted)**					
Mean (SD)	2,075.3 (802.2)	2,463.4 (833.7)	2,086.4 (765.9)	1,654.3 (560.0)	<.001
**Survey cycle**					
2009–2010	15.7	17.1	20.5	9.1	<.001
2011–2012	16.2	17.0	13.5	18.1	
2013–2014	16.5	18.4	11.8	19.7	
2015–2016	16.7	19.3	11.9	19.1	
2017–2020	17.2	17.7	17.0	17.0	
2021–2023	17.6	10.4	25.3	17.0	

Abbreviation: NHANES, National Health and Nutrition Examination Survey.

a Adherence was assessed using the mean of two 24-hour dietary recalls. Fiber was scored positively, and saturated fat and cholesterol were reverse-scored; component scores were summed (range, 0–6), with higher scores indicating greater adherence.

b Values are survey-weighted percentages unless otherwise indicated. Sample sizes are unweighted. *P* values account for the NHANES complex survey design. Percentages for categorical variables were calculated from nonmissing observations; missing percentages are shown separately.

c *P* values were determined using the Pearson χ² test with Rao–Scott adjustment for categorical variables and the design-based Kruskal–Wallis test for continuous variables.

d Body mass index was calculated from measured height and weight as weight in kilograms divided by height in meters squared.

e Includes participants categorized by NHANES as “Other Race – Including Multi-Racial.”

Several demographic and lifestyle characteristics differed across tertiles of plant-forward dietary score (Table 1), including age, sex, race and ethnicity, physical activity, and total energy intake (all *P* < .001). Obesity prevalence differed significantly across tertiles (*P* < .001), with lower prevalence in the highest adherence group (34.5%) compared with the lowest tertile (41.7%). Mean BMI also differed across tertiles, whereas the prevalence of prediabetes and diabetes did not.

In adjusted survey-weighted regression models (Table 2), greater adherence to the plant-forward dietary pattern was associated with more favorable cardiometabolic outcomes. Compared with the tertile for lowest adherence, moderate adherence was associated with lower BMI (β = −0.70; 95% CI, −1.01 to −0.39) and lower odds of obesity (adjusted odds ratio [AOR] = 0.87; 95% CI, 0.79–0.97). High adherence was associated with lower BMI (β = −1.45; 95% CI, −1.82 to −1.09), 30% lower odds of obesity (AOR = 0.70; 95% CI, 0.62–0.79), and 28% lower odds of diabetes (AOR = 0.72; 95% CI, 0.62–0.84). Associations with prediabetes were not significant. Graded associations across tertiles showed no clear trend for prediabetes (Figure).

**Table 2:** Adjusted Associations Between Plant-Forward Dietary Score^a^ and Cardiometabolic Outcomes Among Survey Participants (N = 33,532), NHANES, 2009–2023

Covariate	BMI, β (95% CI)	Obesity, AOR (95% CI)	Prediabetes, AOR (95% CI)	Diabetes, AOR (95% CI)
**Tertile of plant-forward dietary score^a^**				
Low adherence	1 [Reference]	1 [Reference]	1 [Reference]	1 [Reference]
Moderate adherence	−0.70^b^ (−1.01 to −0.39)	0.87^c^ (0.79 to 0.97)	0.91 (0.82 to 1.02)	0.86 (0.74 to 1.00)
High adherence	−1.45^b^ (−1.82 to −1.09)	0.70^b^ (0.62 to 0.79)	0.92 (0.82 to 1.04)	0.72^b^ (0.62 to 0.84)
**Food security level**				
Very low	1.42^b^ (0.81 to 2.02)	1.36^b^ (1.18 to 1.57)	1.01 (0.84 to 1.20)	1.55^b^ (1.26 to 1.90)
Low	1.19^b^ (0.66 to 1.72)	1.34^b^ (1.17 to 1.53)	1.16 (1.00 to 1.35)	1.27^c^ (1.02 to 1.59)
Marginal	0.69^d^ (0.18 to 1.20)	1.23^d^ (1.08 to 1.40)	1.07 (0.90 to 1.28)	1.28^d^ (1.07 to 1.53)
High	1 [Reference]	1 [Reference]	1 [Reference]	1 [Reference]
**Age group, y**				
18–29	1 [Reference]	1 [Reference]	1 [Reference]	1 [Reference]
30–49	2.15^b^ (1.80 to 2.49)	1.59^b^ (1.41 to 1.80)	2.90^b^ (2.45 to 3.44)	4.32^b^ (3.02 to 6.17)
50–64	2.27^b^ (1.86 to 2.68)	1.78^b^ (1.54 to 2.06)	6.20^b^ (5.24 to 7.35)	14.89^b^ (10.76 to 20.60)
≥65	1.37^b^ (0.89 to 1.85)	1.38^b^ (1.18 to 1.62)	8.81^b^ (7.31 to 10.61)	22.02^b^ (15.87 to 30.55)
**Sex**				
Female	0.14 (−0.10 to 0.39)	1.02 (0.93 to 1.12)	1.06 (0.96 to 1.18)	0.70^b^ (0.63 to 0.78)
Male	1 [Reference]	1 [Reference]	1 [Reference]	1 [Reference]
**Race and ethnicity**				
Mexican or Hispanic	1 [Reference]	1 [Reference]	1 [Reference]	1 [Reference]
Non-Hispanic Black	0.97^b^ (0.44 to 1.50)	1.20^d^ (1.06 to 1.37)	1.61^b^ (1.39 to 1.85)	1.01 (0.85 to 1.20)
Non-Hispanic White	−0.64^d^ (−1.07 to −0.21)	0.77^b^ (0.69 to 0.85)	0.70^b^ (0.61 to 0.81)	0.55^b^ (0.47 to 0.64)
Other/multiracial	−2.43^b^ (−2.90 to −1.96)	0.47^b^ (0.40 to 0.55)	1.15 (0.99 to 1.33)	0.87 (0.73 to 1.03)
**Poverty-to-income ratio**				
First tertile (lower income relative to poverty threshold)	1 [Reference]	1 [Reference]	1 [Reference]	1 [Reference]
Second tertile	0.36^c^ (0.04 to 0.68)	1.11^c^ (1.01 to 1.22)	1.02 (0.89 to 1.17)	0.93 (0.81 to 1.07)
Third tertile (higher income relative to poverty threshold)	0.01 (−0.36 to 0.38)	1.02 (0.91 to 1.14)	0.84^c^ (0.72 to 0.96)	0.75^b^ (0.65 to 0.88)
**Physical activity in past week in minutes per week**				
0	1 [Reference]	1 [Reference]	1 [Reference]	1 [Reference]
<150	−0.75^b^ (−1.15 to −0.35)	0.86^d^ (0.77 to 0.96)	0.95 (0.82 to 1.09)	0.80^c^ (0.67 to 0.96)
150–299	−1.78^b^ (−2.21 to −1.35)	0.61^b^ (0.54 to 0.69)	0.88 (0.75 to 1.03)	0.66^b^ (0.54 to 0.80)
≥300	−2.45^b^ (−2.79 to −2.11)	0.53^b^ (0.48 to 0.59)	0.86^c^ (0.74 to 0.98)	0.53^b^ (0.45 to 0.63)
**Energy intake per 100 kcal per day**	−0.03^b^ (−0.05 to −0.02)	0.99^b^ (0.98 to 0.99)	1.01^b^ (1.01 to 1.02)	0.98^b^ (0.97 to 0.98)
**Survey cycle**				
2009–2010	1 [Reference]	1 [Reference]	1 [Reference]	1 [Reference]
2011–2012	0.06 (−0.40 to 0.52)	0.92 (0.80 to 1.06)	0.89 (0.77 to 1.03)	1.10 (0.96 to 1.27)
2013–2014	0.46 (−0.07 to 0.99)	1.05 (0.90 to 1.22)	0.79^b^ (0.69 to 0.90)	1.04 (0.89 to 1.22)
2015–2016	0.76^d ^(0.21 to 1.32)	1.17^c^ (1.01 to 1.36)	0.88^c^ (0.77 to 1.00)	1.23^c^ (1.02 to 1.49)
2017–2020	1.08^b^ (0.68 to 1.49)	1.33^b^ (1.16 to 1.52)	0.76^b^ (0.66 to 0.88)	1.35^b^ (1.20 to 1.51)
2021–2023	1.65^b^ (1.10 to 2.21)	1.38^b^ (1.17 to 1.62)	0.76^d^ (0.65 to 0.90)	1.51^b^ (1.26 to 1.82)

Abbreviation: AOR, adjusted odds ratio; NHANES, National Health and Nutrition Examination Survey.

a Adherence was assessed using the mean of two 24-hour dietary recalls. Fiber was scored positively, and saturated fat and cholesterol were reverse-scored; component scores were summed (range, 0–6), with higher scores indicating greater adherence.

b *P* < .001.

c *P* < .05.

d *P* < .01.

**Figure:**
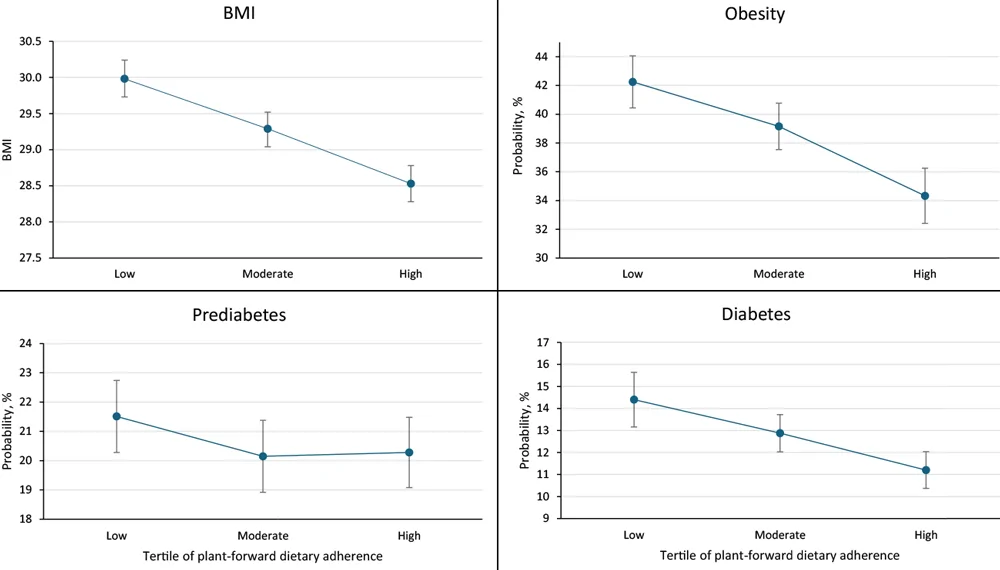
Adjusted cardiometabolic outcomes across tertiles of plant-forward dietary score. Points represent adjusted mean BMI and adjusted predicted probabilities of obesity, prediabetes, and diabetes among participants with low, moderate, and high adherence to the plant-forward dietary pattern, NHANES, 2009–2023. Error bars indicate 95% CIs. Estimates were derived from survey-weighted multivariable regression models adjusted for age, sex, race and ethnicity, poverty-to-income ratio, food security, physical activity, total energy intake, and NHANES survey cycle. Abbreviations: BMI, body mass index, NHANES, National Health and Nutrition Examination Survey.

**Table:** 

Outcome	Tertile	Predicted (95% CI)
BMI	Low	29.98 (29.73–30.24)
	Moderate	29.29 (29.05–29.52)
	High	28.53 (28.28–28.78)

Diabetes, %	Low	14.4 (13.16–15.64)
	Moderate	12.88 (12.03–13.72)
	High	11.2 (10.37–12.04)

Obesity, %	Low	42.25 (40.44–44.06)
	Moderate	39.16 (37.54–40.77)
	High	34.33 (32.41–36.25)

Prediabetes, %	Low	21.51 (20.28–22.74)
	Moderate	20.15 (18.92–21.38)
	High	20.28 (19.08–21.48)

## Discussion

In this nationally representative sample of US adults, greater adherence to the plant-forward dietary pattern was associated with lower BMI and reduced odds of obesity and diabetes. These findings support the hypothesis that nutrient profiles consistent with plant-forward dietary patterns are associated with favorable cardiometabolic health. In contrast, no significant association was observed with prediabetes, which may reflect heterogeneous mechanisms underlying early dysglycemia or limitations of cross-sectional assessment.

These findings are consistent with prior observational and clinical research linking plant-forward and related plant-based dietary patterns to lower body weight and improved cardiometabolic risk profiles (4,8). However, intake of minimally processed plant foods remains relatively low among many US adults (9). Such diets are typically higher in dietary fiber and often, although not universally, lower in saturated fat, characteristics associated with improved metabolic health (8). In contrast, dietary patterns high in red and processed meat have been linked to increased risk of obesity, type 2 diabetes, and other cardiometabolic conditions (10).

From a public health perspective, these findings highlight the potential role of plant-forward dietary patterns in improving cardiometabolic health. Programs such as the Gus Schumacher Nutrition Incentive Program, Food Is Medicine initiatives, and the Special Supplemental Nutrition Program for Women, Infants, and Children, including its Cash Value Benefit for fruits and vegetables, may improve access to minimally processed plant foods through financial incentives and health care–based interventions (11–13). Complementary nutrition education, alongside expanded nutrition assistance initiatives, may support sustained dietary behavior change and improve diet quality, particularly among populations facing structural barriers to healthy food access.

Strengths of this study include the use of nationally representative data with appropriate survey weighting, allowing findings to be generalized to the US noninstitutionalized adult population. Pooling multiple NHANES cycles increased statistical power and stability of estimates, and modeling dietary adherence as tertiles enabled assessment of graded associations.

This study also has several limitations. The cross-sectional design precludes causal inference, and reverse causation cannot be ruled out. Dietary intake was assessed by using self-reported 24-hour dietary recalls, which are subject to measurement error and reporting bias. Residual confounding from unmeasured or imperfectly measured factors cannot be ruled out. Although the analysis used nationally representative data, some subgroup estimates were less precise than others, which warrants cautious interpretation. Finally, the exposure was a study-specific nutrient-derived proxy rather than a validated, food-based, plant-forward dietary index, which may have resulted in some exposure misclassification.

Greater adherence to the plant-forward dietary pattern was associated with more favorable cardiometabolic outcomes. These findings support public health strategies that promote dietary patterns emphasizing minimally processed plant foods as part of broader efforts to improve cardiometabolic health.
